# A variable stiffness robotically steerable guidewire for endovascular interventions

**DOI:** 10.1038/s44182-025-00029-0

**Published:** 2025-07-10

**Authors:** Timothy A. Brumfiel, Revanth Konda, Nidhi Malhotra, Jaydev P. Desai

**Affiliations:** Medical Robotics and Automation (RoboMed) Laboratory, Wallace H. Coulter Department of Biomedical Engineering, Georgia Institute of Technology, Atlanta, GA USA

**Keywords:** Biomedical engineering, Mechanical engineering

## Abstract

Endovascular interventions typically begin with the placement of a guidewire. Guidewire placement is challenging due to tortuous anatomy and the lack of steerability at the guidewire tip. Navigation often requires several guidewires with different stiffnesses to ensure the target is safely reached. This results in longer procedure times, extended radiation exposure to patients, and higher healthcare costs. To address these challenges, we present the design, modeling, and control of a tendon-driven robotically steerable guidewire with controllable stiffness along its proximal segment through a proposed motion control scheme. Models to capture the motion of the guidewire are presented and image feedback is utilized to achieve closed-loop control. The proposed controller exhibited maximum deflection RMSE of 1.82° and 0.70° for the distal and stiffening joints, respectively. The stiffening joint achieved the desired stiffnesses with a maximum RMSE of 1.9 × 10^−2^ Nm^2^. Thus, the methods presented in this paper demonstrate the potential to use a single guidewire in a procedure.

## Introducton

**Fig. 1:**
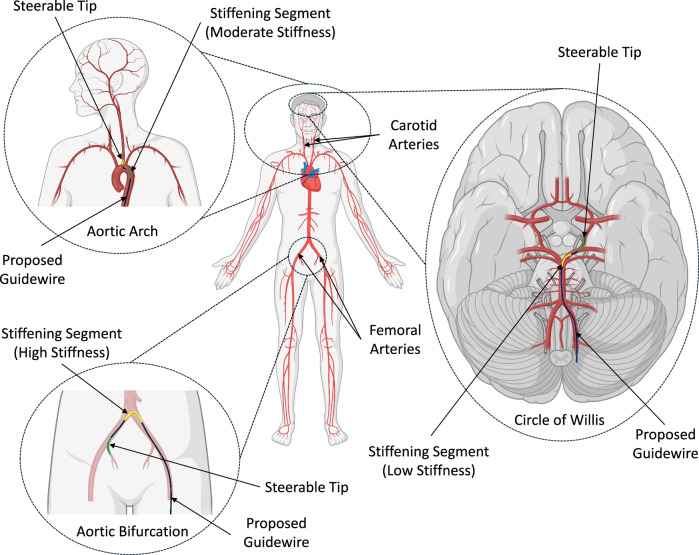
Overview of potential applications of the proposed robotically steerable guidewire. A schematic depicting potential applications of the proposed robotically steerable guidewire. The steerable tip and the stiffening segment of the guidewire are highlighted in green and yellow colors, respectively. Created in BioRender. Konda, R. (2024) BioRender.com/q67y818.

Cardiovascular diseases (CVDs) are medical conditions pertaining to the heart or blood vessels, accounting for 32% of deaths globally in 2019^1^. Minimally invasive treatments, namely endovascular interventions for CVDs, begin with the placement of a long slender wire, called a guidewire, to a target area in the vasculature to serve as a guide for the placement of other devices such as stents and catheters^2,3^. Currently, guidewires have diameters in the range of 0.008^″^–0.038^″^ (0.20 mm–0.965 mm) and are manually steered to the target area within the vasculature by a skilled clinician^4^. The manual placement of the guidewire is challenging due to the lack of active steerability at the tip of the guidewire, potentially resulting in guidewire fracture^5^ or vessel perforation^6^. Furthermore, in procedures requiring the traversal of highly tortuous vasculatures, it is a common practice to utilize several different guidewires to provide the appropriate stiffness in different sections of the blood vessels^4^. For example, in the treatment of stroke, a clinician may utilize a 0.035^″^ guidewire within the femoral artery (Fig. 1) and transition to a 0.014^″^ guidewire when accessing past the carotid artery (Fig. 1) toward the vasculatures in the brain^7^. Certain procedures also utilize a microcatheter to provide additional support to the proximal end of the guidewire to facilitate efficient navigation^8,9^. These type of techniques lead to longer procedure times and extended radiation exposure to the patient and the clinical staff, in addition to higher healthcare costs.

Continuum robots, characterized by continuously deformable structures, have shown promise in addressing challenges in minimally invasive surgery^10^. These robots are particularly useful due to their inherent compliance, ability to provide contact force measurements from shape^11,12^, and high potential for miniaturization. A robotically steerable guidewire is an example of a continuum robot with a sub-mm outer diameter (OD), primarily actuated using either a tendon-driven mechanism^4,13^ or a magnetically actuated mechanism^8,14–16^. Among these, tendon-driven guidewires have the benefit of compact actuation mechanisms and minimal additional equipment compared to magnetically actuated guidewires. By demonstrating different functionalities including complex motion capabilities^4,13,17–20^, integration of force and shape sensing^11,12,21,22^, and acceptable control performances^23,24^, tendon-driven robotically steerable guidewires have exhibited great potential for applications in endovascular interventions. However, due to their inherent compliance, these guidewires may still require additional support at their proximal end (away from the steerable tip) to enable efficient navigation. Furthermore, due to their fixed stiffnesses, these guidewires may not be suitable for navigation in different vessel anatomies throughout the human body for a given procedure.

Previous studies on surgical robots have utilized “shape locking” mechanisms at the proximal end of the device to further enhance its structural stability and aid in manipulation at the tip^25,26^. Mechanisms such as baskets and balloons, have been predominantly utilized for this purpose^26–29^. However, most of these mechanisms are designed to use contact with the inner walls of the vessels^25^. Although this strategy may result in efficient stabilization of the robot, each method can potentially cause damage to the tissue during interactions with the vessel walls. For example, studies have shown that basket mechanisms can cause notable damage to the walls of the blood vessels^30^ and that balloon mechanisms often result in angiographic dissection^31^. Furthermore, due to the size constraints of the guidewire, integration of these mechanisms into the structure of the guidewire can be highly challenging. The introduction of variable stiffness capabilities at the proximal end of the guidewire may facilitate efficient navigation of the guidewire without using additional structures. Furthermore, due to the ability to vary stiffness, these type of guidewires may be able to navigate through different vessel anatomies without the need for switching guidewires for a given procedure^32^. Examples of potential applications for robotically steerable guidewires with stiffness variation capabilities include peripheral access, valve implant, and neuroendovascular interventions, as presented in Fig. 1.

Variable stiffness is a popular phenomenon demonstrated in many continuum robotic systems^33–35^. Several methods to achieve variable stiffness have been explored with popular strategies being antagonism, jamming, and usage of thermally responsive materials, among others^35^. Jamming involves increasing the stiffness of a segment of the continuum robot by increasing the friction between an introduced medium and the compliant structure such that it behaves in a rigid manner. Examples of jamming include increasing friction between fibers through pressurized air^36,37^, introducing additional material such as granular particles or fluids^38,39^, or by utilizing layer jamming flaps^40^. Another popular stiffening approach is the usage of thermally responsive materials whose mechanical properties can be varied by controlling the temperature applied to them^41–43^. However, the aforementioned approaches may not be physically feasible in robotically steerable guidewires due to their sub-mm OD. Antagonism is a stiffening approach in which the stiffness of the continuum robot is varied by utilizing opposing forces^35^. The opposing forces to realize stiffness variation have been achieved through the use of tendons^44,45^, fluidic actuators^46,47^, or a combination of both^48,49^ in existing literature. Stiffness variation through fluidic antagonism may not be suitable for employment in robotically steerable guidewires due to tight constraints on size of the guidewire and the space requirements of the fluidic system to enable stiffening capabilities. However, stiffness variation through tendon actuation can be suitable for application in robotically steerable guidewires. Meanwhile, integration of additional tendons into the body of the guidewire presents unique challenges due to the sub-mm OD requirements of guidewires.

In our previous work, we developed the COaxially Aligned STeerable (COAST) guidewire robot^4^. The COAST guidewire robot comprises three coaxially aligned nitinol tubes: the inner, middle, and outer tube. The middle tube, laser micromachined with a unidirectional asymmetric notch (UAN) pattern, is actively bent in a single plane by a nitinol tendon. The unmachined inner tube can be translated relative to the middle tube, varying stiffness in the joint significantly. Unlike traditional notched continuum robots, the relative translation of the inner tube allows for the length of the bending segment to be varied. Lastly, the outer tube of the COAST guidewire robot is laser micromachined with a UAN pattern and is capable of translation relative to the middle tube, allowing the guidewire tip to be advanced forward along the tangent at the distal end of the middle tube. This configuration of tubes allows for follow-the-leader (FTL) motion, where the body of the robot follows the path taken by the tip, which can allow for blood vessel traversal with minimal wall contact. However, the robotic guidewire presented by Jeong et al.^4^ is only capable of bending in a single direction within a plane. Furthermore, while relative rotation of the middle and outer tubes can vary the stiffness of the robot^18^, there is a direct trade-off between steerability and stiffness control (i.e., high stiffness configurations allow minimal joint deflection).

In this work, we present the design of a robotically steerable guidewire capable of achieving a desired stiffness at its proximal end through motion control of the steerable proximal segment. The design utilizes a nitinol tube machined with two notched segments: (1) The first segment with a tendon attached at the distal end of the tube, is the steerable tip of the guidewire; (2) The second segment attached with two independently controlled tendons at the proximal end of the tube (away from the distal tip), is the stiffening segment of the guidewire. While unable to achieve FTL motion, this design enables “S”-shaped curves within a plane, minimal occlusion of the inner lumen of the guidewire as a result of routing tendons on the exterior, and allows for stiffness control and steerability to be independent tasks. To systematically estimate the behavior of the proposed guidewire comprising the coupled motion of the two segments, a detailed physics-based model is presented. Additionally, image feedback, in combination with inverse models, is utilized to control the pose and stiffening capabilities of the guidewire in a closed-loop manner. The primary contributions of this work are:

- Design of a sub-mm tendon-driven robotically steerable guidewire with an active stiffening segment.
- Modeling of the kinematic behavior of the proposed two degrees-of-freedom variable stiffness guidewire.
- Image-based closed-loop control of the joint angles and stiffness tuning of the proposed variable stiffness guidewire through motion control.

The paper is organized as follows: In the ‘Results’ section, we first present the ‘Proposed Design’ and ‘Actuation Mechanism’ subsections which detail the design of the proposed robotically steerable guidewire with active stiffening capabilities and the actuation mechanism. Secondly, we detail the image feedback utilized in each experiment in the ‘Image Feedback’ subsection. Thirdly, we present the ‘Model Validation’ subsection, experimentally validating the mechanics models developed, the ‘Guidewire Joint Control’ subsection, evaluating the joint control scheme utilized in this work, and the ‘Stiffening Validation’ subsection, which evaluates a stiffening control law to demonstrate the stiffness tuning capabilities of the proposed robotically steerable guidewire. We further demonstrate the motion control approach in a phantom aortic arch in the ‘Demonstration in Phantom Vasculature’ subsection. We next present a thorough discussion of the results presented in the ‘Discussion’ section. Lastly, the ‘Methods’ section is presented, detailing the mechanics models and the control scheme used for active stiffness control.

## Results

### Proposed design

**Fig. 2:**
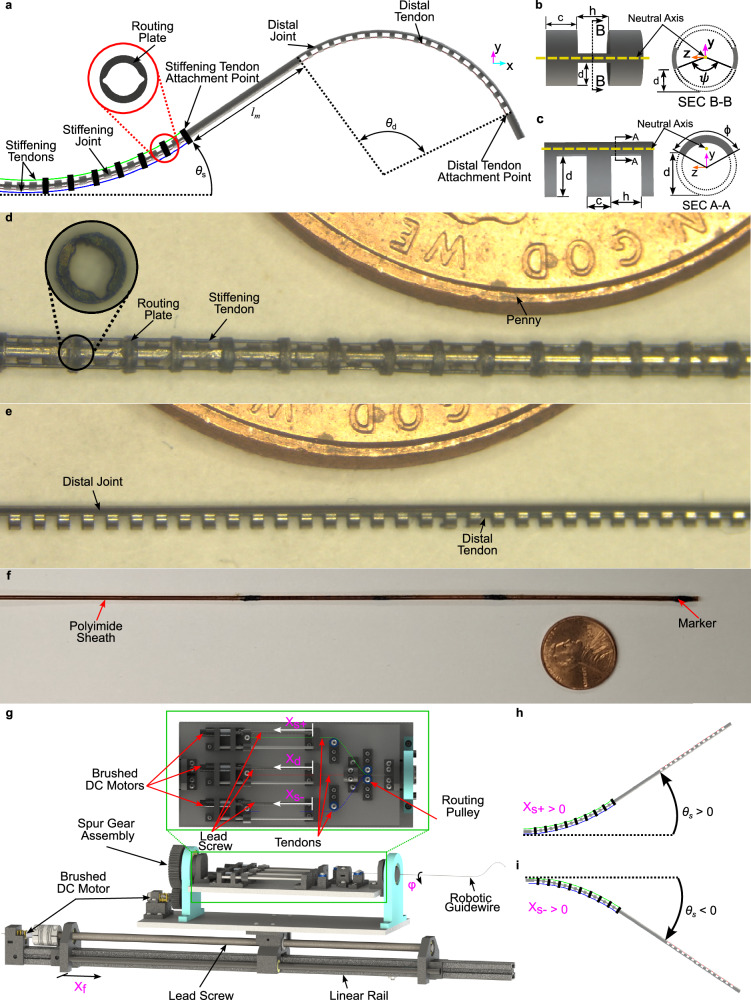
Overview of design of the proposed robotically steerable guidewire and the actuation system. A schematic of **a** the robotically steerable guidewire developed in this work, **b** parameters of a BSN pattern, **c** parameters of a UAN pattern. An image of the **d** stiffening joint, **e** distal joint, and **f** the robotically steerable guidewire in a polyimide sheath. A schematic of **g** the actuation system, **h** resulting stiffening segment motion from *X*_*s*+_, and **i** resulting stiffening segment motion from *X*_*s*−_.

The robotically steerable guidewire developed in this work, shown in Fig. 2a, comprises a 0.62 mm OD nitinol tube (Edgetech Industries LLC, FL, USA) that is laser micromachined on a femtosecond laser (Optec Laser S.A., Frameries, Belgium) at two locations along the length. During laser micromachining, the laser beam is always radially aligned and the tube is rotated to cut out an arc along the circumference while being translated longitudinally, if necessary, for the cut geometry. The proximal segment, hereby referred to as the stiffening joint, comprises a bidirectional symmetric notch (BSN) pattern as shown in Fig. 2b. The distal segment of the guidewire, hereby referred to as the distal joint, comprises a UAN pattern as shown in Fig. 2c. The aforementioned notch patterns provide the corresponding joints with the ability to control their deflection in the plane in which the notches have been machined. Stiffness variation is achieved by systematically controlling the deflection of the stiffening joint. Depending on the location of the vessel, the desired stiffness of the stiffening joint may vary. For example, while traversing the aortic bifurcation, high stiffness is often desired at the location of the bifurcation to prevent the guidewire from “climbing” up the abdominal aorta^50^. Similarly, after traversing the aorta and entering the carotid arteries through the aortic arch, moderate stiffness of the guidewire may be desired. For navigation in neurovasculature, low stiffness of the stiffening joint is predominantly preferred^7^. Furthermore, due to the design of the proposed guidewire, motion coupling of the two joints is expected^51^. High stiffness of the stiffening joint would lead to minimal motion coupling of the two joints, whereas lower stiffness of the stiffening joint would lead to considerable motion coupling of the two joints. Additionally, the stiffening joint can also be controlled to achieve a desired bending angle. Consequently, the proposed robotically steerable guidewire can be controlled to achieve “S”-shapes and “J”-shapes with variable curvatures using the distal and stiffening joints.

Table 1 presents a comprehensive list defining each symbol utilized in this work. Each joint is characterized by the depth of cut, *d*, the notch spacing, *c*, and the notch width, *h*, as shown in Fig. 2b, c for the BSN and UAN patterns, respectively. The distal joint and stiffening joint comprise *N*_*d*_ and *N*_*s*_ notches, respectively. The UAN pattern is characterized by the angle of material remaining after laser micromachining, *ϕ*, given by:

1 $$\phi =\left\{\begin{array}{ll}2\arccos \left(\frac{{d}_{d}-{r}_{o}}{{r}_{o}}\right)\quad &\,{\text{if}}\,\ \ {d}_{d} \,>\, {r}_{o}\\ 2\pi -2\arccos \left(\frac{{r}_{o}-{d}_{d}}{{r}_{o}}\right)\quad &\,{\text{if}}\,\ \ {d}_{d} \,<\, {r}_{o}\\ \pi\hfill \quad \quad \quad \quad &\,{\text{if}}\,\ \ {d}_{d}={r}_{o}\end{array}\right.$$

where *d*_*d*_ is the depth of cut of the distal joint, and *r*_*o*_ is the outer radius of the tube. The BSN pattern is characterized by the angle of material removed, $\psi$, given by:

2 $$\psi =2\arccos \left(\frac{{r}_{o}-{d}_{s}}{{r}_{o}}\right),\quad \,{\text{where}}\,:\quad {d}_{s}\in ({r}_{o}-{r}_{i},{r}_{o})$$

where *d*_*s*_ is the depth of cut of the stiffening joint, and *r*_*i*_ is the inner radius of the tube. The distal joint is actuated by a nitinol tendon (0.152 mm OD) attached to the inner wall of the tube at the tip of the guidewire using J-B Weld KwikWeld Steel Reinforced Epoxy (J-B Weld, Sulphur Springs, TX, USA) and is routed through the lumen of the tube. The stiffening joint is actuated by a pair of independently controlled tendons (0.076 mm OD), which are attached to a nitinol ring that is placed at the distal end of the stiffening joint using epoxy and routed along the outer surface of the tube. This was done to prevent unwanted interactions between the tendons actuating the two joints. Several laser micromachined rings termed as “routing plates”, shown in Fig. 2a (inset) are secured on the outer surface of the stiffening joint to route the tendons such that the tendons follow the curvature of the stiffening joint. The stiffening and distal joints are separated by a small unmachined segment of length *l*_*m*_ to separate the two joint segments and increase the workspace of the robotic guidewire. An image of the stiffening joint and the distal joint of the proposed robotically steerable guidewire is shown in Fig. 2d, e, respectively. The length of the prototype presented in this work is 749.3 mm. It is possible to have a longer prototype, however, this would result in increased friction. The length of each notched segment was selected to be able to traverse the femoral artery^52^, a common location for the treatment of conditions such as peripheral artery disease, which may use a guidewire of OD similar to that developed in this work. As a result, the stiffening segment and distal segment lengths were selected to both be 35.72 mm, whereas the intermediate unmachined segment was selected to have a 20 mm length. The guidewire is enclosed in a polyimide sheath (Zeus Company LLC, SC, USA), shown in Fig. 2f, with an inner radius and wall thickness of 0.02^*″*^ and 0.001^*″*^, respectively. The guidewire is attached to the polyimide sheath at the tip and the base using epoxy. The sheath is used to prevent fluid from traveling through the lumen of the guidewire under the intended operating conditions and to further constrain the stiffening joint tendons to the outer surface of the guidewire body. Biocompatible and hydrophobic materials such as Pebax® or PTFE could be used as less stiff alternatives to polyimide, resulting in a larger workspace for the same actuation force range. By incorporating the routing plates and the sheath to enable the proposed active stiffening approach, we measured the largest section of the guidewire to be approximately 1.06 mm (~0.042^*″*^).

**Table 1:** Mathematical symbols

Symbol	Meaning	Units
*d*	Notch depth of cut	m
*d*_*d*_	Distal joint notch depth of cut	m
*d*_*s*_	Stiffening joint notch depth of cut	m
*c*	Notch spacing	m
*h*	Notch width	m
*N*_*d*_	Number of distal joint notches	–
*N*_*s*_	Number of stiffening joint notches	–
*r*_*o*_	Nitinol tube outer radius	m
*r*_*i*_	Nitinol tube inner radius	m
*ϕ*	Angle of material remaining after laser micromaching for a UAN pattern	radians
$\psi$	Angle of material removed after laser micromaching for a BSN pattern	radians
*l*_*s*_	Stiffening joint length	m
*l*_*d*_	Distal joint length	m
*l*_*m*_	Unmachined length between the stiffening and distal joint	m
*θ*_*s*_	Total deflection of the stiffening joint	radians
*θ*_*d*_	Total deflection of the distal joint	radians
*φ*	Actuation mechanism roll angle	radians
*X*_*d*_	Distal joint tendon stroke	m
*X*_*s*_	Stiffening joint tendon stroke	m
*X*_*f*_	Guidewire feed	mm
*E*	Young’s modulus of the nitinol tube	GPa
*I*_*s*_	Second moment of area of the stiffening joint notch	m^4^
*I*_*d*_	Second moment of area of the distal joint notch	m^4^
*I*_*s*_	Second moment of area of the sheath	m^4^
*A*_*s*_	Cross-sectional area of the stiffening joint notch	m^2^
*A*_*d*_	Cross-sectional area of the distal joint notch	m^2^
*E*_*sh*_	Young’s modulus of the sheath,	GPa
*E*_*ts*_	Young’s modulus of the tendon of the stiffening joint	GPa
*E*_*td*_	Young’s modulus of the tendon of the distal joint	GPa
Δ*y*_*d*_	Distance between the neutral axis of the distal joint and the line of action of the distal tendon tension	m
Δ*y*_*s*_	Distance between the neutral axis of the stiffening joint and the line of action of the stiffening tendon tension	m
Δ*y*_*d*,*s*_	Distance between the neutral axis of the stiffening joint and the line of action of the distal tendon tension	m
*r*_*td*_	Distal tendon radius	m
*r*_*ts*_	Stiffening tendon radius	m
*r*_*o*,*sh*_	Outer radius of the polymide sheath	m
*r*_*i*,*sh*_	Inner radius of the polymide sheath	m
*η*_*d*_	Inverse of the friction loss from the distal segment to the actuation system	–
*η*_*s*_	Inverse of the friction loss from the stiffening segment to the actuation system	–
*η*_*d*,*s*_	Inverse of the friction loss from the distal segment to the stiffening segment	–
*α*	Total wrapping angle of tendon pulleys	radians
*μ*	Coefficient of friction between nitinol tendons and pulleys	–
*K*_*des*_	Desired stiffness	Nm^2^
*θ*_*s*,*des*_	Reference stiffening joint deflection	radians
*θ*_*d*,*des*_	Reference distal joint deflection	radians
Δ*θ*_*s*_	Variation in the stiffening joint setpoint resulting from a stiffness control law	radians
*θ*_*s*,*set*_	Stiffening joint deflection setpoint	radians

The variables used throughout the design, analysis, and modeling and the corresponding units.

### Actuation mechanism

The actuation mechanism developed for this work is shown in Fig. 2g. The robotically steerable guidewire is clamped within the front portion of the actuation system. The tendon attached to the distal joint is routed straight to its attachment point on the actuation mechanism, whereas the tendons attached to the stiffening joint are routed through a system of pulleys to their respective attachment points. Each tendon is actuated by a DC motor (Maxon Precision Motors (P/N: 347725), MA, United States) with a 64:1 gear ratio (0.06 Nm nominal torque) connected to a lead screw (0.5 mm/rev). The mechanism rests on a platform connected to a spur gear assembly with a 2:1 gear ratio that is driven by a brushed DC motor (Pololu Robotics and Electronics (P/N 5227), NV, USA) with a 380:1 gear ratio (0.36 Nm nominal torque). This enables 3D motion by rotating the bending plane of the guidewire. The platform is connected to a linear rail comprising a 380:1 gear ratio brushed DC motor (Pololu Robotics and Electronics (P/N 5227), NV, USA) attached to a precision lead screw that enables the translation of the entire guidewire system. The control variables of the mechanism are summarized as:

$$\left[\begin{array}{c}\varphi \\ {X}_{d}\\ {X}_{s}\\ {X}_{f}\end{array}\right]=\left[\begin{array}{c}\,{\text{Roll}}\, {\text{of}}\, {\text{the}}\, {\text{Mechanism}}\,\\ \,{\text{Distal}}\, {\text{Tendon}}\, {\text{Stroke}}\,\\ \,{\text{Stiffening}}\, {\text{Tendon}} {\text{Stroke}}\,\\ \,{\text{Guidewire}}\, {\text{Feed}}\,\end{array}\right]$$

where the stiffening tendon stroke, *X*_*s*_, is given by:

3 $${X}_{s}=\left\{\begin{array}{ll}{X}_{s+}\quad \,{\text{if}}\,\quad sgn\left({\theta }_{s}\right)\ge 0\\ {X}_{s-}\quad \,{\text{otherwise}}\hfill\end{array}\right.{\text{, and}}\,\quad {X}_{s+}=-{X}_{s-}$$

where *θ*_*s*_ is the deflection angle of the constant curvature arc formed by the actuated stiffening joint as shown in Fig. 2a, *X*_*s*+_ is the stroke of the tendon that results in a positive bending angle of the stiffening segment (shown in Fig. 2h), and *X*_*s*−_ is the stroke of the tendon that results in a negative bending angle of the stiffening segment (shown in Fig. 2i). Similarly, *θ*_*d*_ is the deflection angle of the constant curvature arc formed by the actuated distal joint as shown in Fig. 2a. As indicated in Eq. (3), the stiffening joint tendons are actuated independently such that only one tendon applies a moment to the stiffening joint. In the event that both tendons are loaded simultaneously, the moment contribution of both tendons would need to be considered.

### Experimental setup

**Fig. 3:**
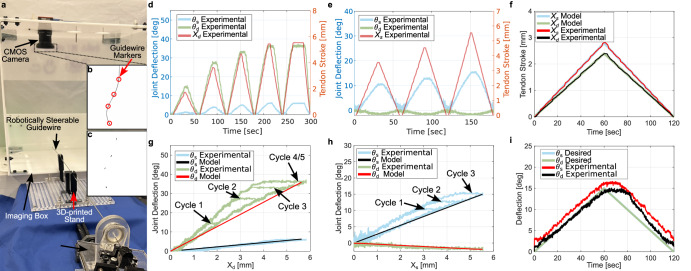
Model validation results. **a** The experimental setup to validate model performance, **b** an image from the CMOS camera to track the guidewire markers, and **c** the segmented markers. The tendon stroke and joint deflection profiles for **d** only distal joint actuation, **e** only stiffening joint actuation, and **f** the actuation of both joints. The experimental and modeled deflection versus tendon stroke values for **g** only distal joint actuation, **h** only stiffening joint actuation, and **i** the modeled and measured deflection for bending both joints simultaneously.

The experimental setup utilized for the model and control algorithm validation, shown in Fig. 3a, comprised of a rectangular box, fabricated from acrylic, to hold a camera. A box was used in place of a simple stand to ensure adequate lighting for efficiently obtaining imaging feedback from the camera. The box was designed such that one of the lateral faces was open – this opening was used to place the guidewire in position under the camera. The top face of the box comprised a hole and attachment mechanism to hold the camera approximately parallel to the base. In this work, a CMOS camera (Zelux® 1.6 MP, Thorlabs Inc., NJ, USA) is utilized to gather gray-scale images of the robotically steerable guidewire. The unmachined segment of the guidewire behind the stiffening joint was supported by a straight stainless-steel tube held in 3D-printed stands. This was done to emulate scenarios where the guidewire is constrained in the vessel along its length. Furthermore, the supporting tube ensured that any undesirable motion that occurred due to the long length of the guidewire was minimized.

### Image feedback

To control the different motion capabilities of the proposed robotically steerable guidewire in a closed-loop manner, image feedback is used. Black markers (or fiducials) are painted on the sheath with their centroids approximately located at the beginning and the end of each notched section as shown in Fig. 3b. Each marker is intended to act in place of a radiopaque marker that would be utilized under fluoroscopy, a common imaging modality used in endovascular interventions. Similar markers could be constructed by plating the guidewire with biocompatible and radiopaque materials such as gold, tungsten, or platinum. Once the image is acquired, the centroids of the markers are determined using the MATLAB Image Processing Toolbox^TM^ (MathWorks, Natick, MA, USA) as shown in Fig. 3c. The orientation of each segment is determined using singular value decomposition (SVD) of the segmented marker, taking the angle of the major axis of the ellipse fit to the segments, and measuring the displacement from the imaging *x*-axis. The angle of each joint is determined by the difference in the distal and proximal markers for the joint. This angle can then be utilized for closed-loop control.

### Model validation

To validate the model derived in the ‘Methods’ section, the guidewire was given different tendon stroke profiles and imaged from above with the camera. Fig. 3d shows the measured angles and distal tendon profiles used to actuate the guidewire with only the distal tendon. The distal joint underwent five 60 second loading and unloading cycles with increasing maximum tendon stroke. Fig. 3e shows the measured angles and distal tendon profiles used to actuate the guidewire with only the stiffening joint tendons. Similar to the distal joint, the stiffening joint underwent three 60 second loading and unloading cycles with increasing maximum tendon stroke. The observed motion of the distal joint, when only actuation of the stiffening segment occurs, is the result of the slacked distal tendon pushing on the distal joint. Lastly, Fig. 3f shows the measured and modeled tendon stroke required to achieve 20° deflection of both joints simultaneously. Fig. 3g shows the joint deflections versus the distal tendon stroke. Hysteretic behavior is observed in the *X*_*d*_ − *θ*_*d*_ relationship. The plateau in the loading and unloading curves is hypothesized to be due to static friction between the nitinol tendon and the tube. The friction between the tendon and the tube transitions from static to dynamic friction resulting in sliding behavior when the unloading curve begins. This hypothesis is reinforced by the data presented in Fig. 3d where a plateau in the distal angle during a monotonically increasing tendon stroke would imply the nitinol tendon entering the superelastic regime. Furthermore, a phase transition of the material itself would be present in Fig. 3g as a piecewise linear function with slopes differing while in the austenite and martensite phases of nitinol. While this friction-induced hysteretic phenomena can be characterized as a non-linear function of the tendon stroke^11^, in this work we will use image feedback to compensate for these effects. As a result, the model is calibrated against the loading curve of the data. For the data presented in Fig. 3g, the model for the distal and stiffening joints contains a maximum error of 3.12° and 0.80°, respectively.

Similarly, the stiffening joint was actuated with the measured deflections and tendon stroke being shown in Fig. 3e. The resulting deflection versus tendon stroke relationship is shown in Fig. 3h along with the model. The model for the distal and stiffening joints results in a maximum error of 0.28° and 1.13°, respectively. The experimental and modeled performance when both joints are simultaneously actuated is shown in Fig. 3i. The model deviates from the measured values most significantly when unloading occurs due to the aforementioned nonlinearities. However, the accuracy of these models was deemed to be acceptable for closed-loop control.

### Guidewire joint control

**Fig. 4:**
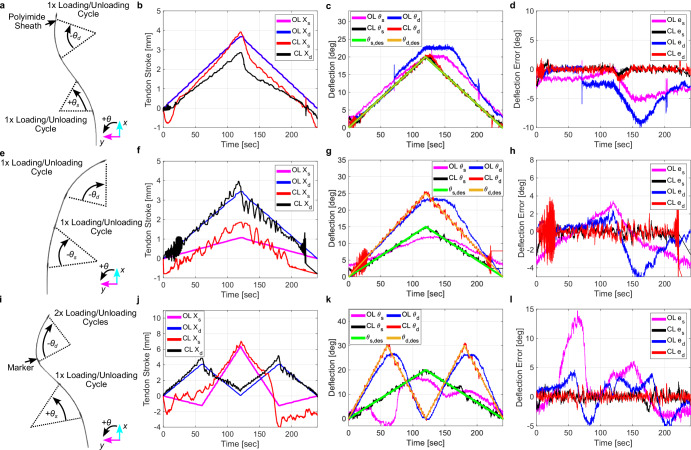
Open-loop and closed-loop control results of the joint angles of the proposed guidewire. The open-loop (OL) and closed-loop (CL) control performance showing **a** an image of the configuration of the robotically steerable guidewire at maximum bending angles with an indicator for the number of loading/unloading cycles, **b** the tendon stroke profiles, **c** the absolute deflection response, and **d** the corresponding deflection errors for trajectory 1. **e** An image of the configuration of the robotically steerable guidewire at maximum bending angles with an indicator for the number of loading/unloading cycles, **f** the tendon stroke profiles, **g** the absolute deflection response, and **h** the corresponding deflection errors for trajectory 2. **i** An image of the configuration of the robotically steerable guidewire at maximum bending angles with an indicator for the number of loading/unloading cycles, **j** the tendon stroke profiles, **k** the absolute deflection response, and **l** the corresponding deflection errors for trajectory 3.

To improve the control of the joint angles of the robotically steerable guidewire in the presence of hysteretic effects, the image feedback was used, in conjunction with the inverse kinematic models, to improve reference angle tracking. A Proportional-Integral-Derivative (PID) controller was used on the joint angle feedback to provide corrections to the tendon stroke, primarily in the unloading phase of a joint trajectory. The PID controller was tuned heuristically. Several reference trajectories and measured absolute joint angles are shown in Fig. 4. Trajectory 1 comprises bending both joints to an absolute angle of 20° as shown in Fig. 4a. The tendon strokes for the first trajectory are shown in Fig. 4b. The corresponding absolute joint deflections and the joint deflection errors are shown in Fig. 4c, d, respectively. For the first trajectory, open-loop control resulted in a RMSE of 3.01° and 4.27° for the stiffening joint and the distal joint, respectively. The closed-loop response resulted in a RMSE of 0.49° and 0.53° for the stiffening joint and the distal joint, respectively.

Trajectory 2 comprises bending the distal joint to an absolute angle of 25° and the stiffening joint to an absolute angle of 15° as shown in Fig. 4e. The tendon strokes for the second trajectory are shown in Fig. 4f. The corresponding joint deflections and the joint deflection errors are shown in Fig. 4g, h, respectively. For the second trajectory, the open-loop control resulted in a RMSE of 1.98° and 2.06° for the stiffening joint and the distal joint, respectively. The closed-loop response resulted in a RMSE of 0.65° and 1.82° for the stiffening joint and the distal joint, respectively.

Trajectory 3 comprises two loading/unloading cycles of the distal joint to an absolute angle of 30° while the stiffening joint undergoes one loading/unloading cycle to an absolute angle of 20° as shown in Fig. 4i. The tendon strokes for the third trajectory are shown in Fig. 4j. The corresponding joint deflections and the joint deflection errors are shown in Fig. 4k and Fig. 4l, respectively. For the third trajectory, the open-loop control resulted in a RMSE of 4.97° and 2.54° for the stiffening joint and the distal joint, respectively. The closed-loop response resulted in a RMSE of 0.70° and 0.59° for the stiffening joint and the distal joint, respectively. The errors are seen to be much higher when the distal joint initially begins the first loading cycle. This is hypothesized to be caused by initial deviations from the model due to the unaccounted hysteretic effects of the distal joint onto the stiffening joint and the selection of a low bandwidth feedback controller. A low bandwidth controller was selected to limit the motion of the robotically steerable guidewire to quasi-static motions. During the second cycle, this phenomena is seen at a much lower amplitude, hypothesized to be the result of a non-zero integral term coming from the PID controller. However, the closed-loop performance is deemed sufficient for tracking a desired deflection profile due to the <1° average tracking error for the tested range of 0° to 45° bending angle for each joint.

The fluctuations observed in Fig. 4b, c are hypothesized to be due to the camera feedback used to determine the joint angles. The segmentation of the markers on the guidewire can vary in performance due to the trajectory occurring or lighting fluctuations, resulting in noise in the angle feedback. The fluctuations observed in Fig. 4f, g are hypothesized to be due to initial tendon engagement inducing slight vibrations. This, in conjunction with actuation mechanism machining inaccuracies, assembly inaccuracies, observed out-of-roundness error in a lead screw, and the camera resolution used for feedback, could have potentially contributed to the measured oscillations and measured errors in the experimental data. Furthermore, the selected values of the PID gains may have also contributed towards the vibrations in the output.

### Stiffening validation

**Fig. 5:**
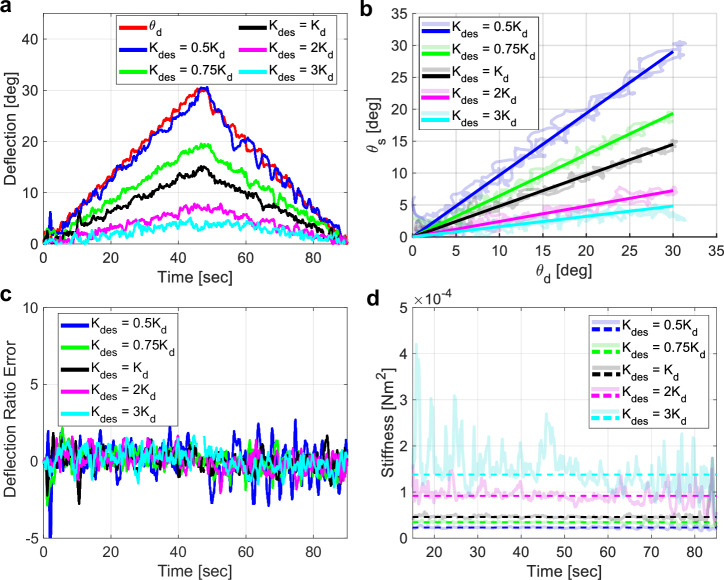
Closed-loop control results of the stiffness tuning capabilities. **a** Trajectories followed for distal joint deflection while achieving a desired stiffness profile of the stiffening joint, **b** the corresponding deflection ratios, **c** the error between theoretical deflection ratios and desired deflection ratios, and **d** the experimental and desired stiffness profiles.

To validate the stiffness tuning capabilities of the robotically steerable guidewire, different joint stiffnesses were determined to achieve a desired joint coupling using the feedback control law presented in the ‘Methods’ section. The scalar multiples of the distal joint stiffness, *K*_*d*_, were used as the desired stiffness of the stiffening joint, *K*_*des*_. These desired stiffness values were used in the feedback control law. For each trajectory for a desired stiffness profile, the stiffening joint was given a desired value of 0° and the distal joint was given a reference that monotonically increases to an angle of 30° followed by monotonically decreasing to an angle of 0°. The time response as the distal joint bends with *K*_*des*_ values of 0.5*K*_*d*_, 0.75*K*_*d*_, *K*_*d*_, 2*K*_*d*_, and 3*K*_*d*_ is shown in Fig. 5a where we can see that as the stiffness is increased, the stiffening joint deflects less throughout the trajectory. Conversely, when the desired proximal stiffness is the same as the distal stiffness (*K*_*des*_ = *K*_*d*_) the deflection of the stiffening joint is approximately half of the distal joint. This is because the moment arm from the neutral axis of the stiffening joint to the line of action of the distal tendon is approximately half that of the moment arm of the distal joint. Fig. 5b shows the measured deflection ratios and the modeled deflection ratios given a desired stiffness for the stiffening joint. Fig. 5c shows the errors between the modeled and measured joint ratios where the RMSE is determined to be 1.10°, 0.67°, 0.59°, 0.6°, and 0.70° for desired stiffness values of 0.5*K*_*d*_, 0.75*K*_*d*_, *K*_*d*_, 2*K*_*d*_, and 3*K*_*d*_, respectively. Higher errors associated with more compliant behavior is hypothesized to be the result of significant deviation from the natural stiffness of the stiffening joint. Furthermore, the experimental and desired stiffness profiles are shown in Fig. 5d. The RMSE between the experimental and desired stiffness is 4.27 × 10^−4^ Nm^2^, 1.13 × 10^−5^ Nm^2^, 7.77 × 10^−4^ Nm^2^, 1.90 × 10^−2^ Nm^2^, and 1.67 × 10^−2^ Nm^2^ for desired stiffness values of 0.5*K*_*d*_, 0.75*K*_*d*_, *K*_*d*_, 2*K*_*d*_, and 3*K*_*d*_, respectively.

### Demonstration in phantom vasculature

**Fig. 6:**
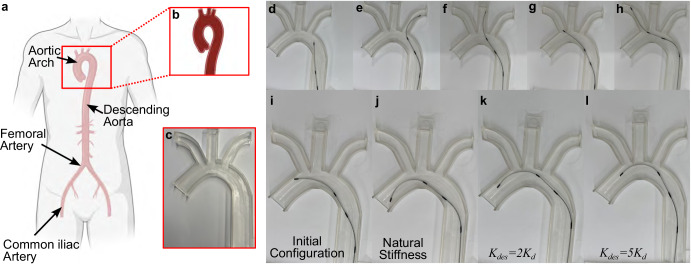
Demonstration of the proposed guidewire in phantom vasculature. **a** A schematic of the blood vessel anatomy, **b** a schematic of the aortic arch, and **c** the 3D-printed phantom used for the demonstration. Traversal through the aortic arch and branches showing **d** introduction of the guidewire into the aortic arch, navigation into the **e** first and **f** second branches, and **g** transition and **h** advancement into the third branch. Active stiffening demonstration across the aortic arch showing **i** the initial configuration followed by advancement and distal bending using **j** the natural stiffness of the stiffening joint, **k** *K*_*des*_ = 2*K*_*d*_, and **l** *K*_*des*_ = 5*K*_*d*_. Created in BioRender. Konda, R. (2025) https://BioRender.com/gdhqbq2.

To demonstrate the navigation of the guidewire in the blood vessels (Fig. 6a) with and without stiffness control, several traversals in a phantom model were conducted. A phantom model of a slice of an aortic arch (Fig. 6b) was 3D-printed (Fig. 6c) for this demonstration. A 35D durometer Pebax® (OD: 1.30 mm (~0.051^*″*^), ID: 1.06 mm (~0.042^*″*^)) with a Young’s modulus of *E*_*sh*_ = 21 MPa (Zeus Company LLC, S.C, USA) was utilized for the demonstration. The guidewire was operated using a remote controller (Xbox 360 Controller, Microsoft, WA) to provide reference commands to each joint. First, to demonstrate the effectiveness of the 2-DoF robotically steerable guidewire, the guidewire was navigated to all peripheral branches of the phantom model. The guidewire was first introduced into the aortic arch through the descending aorta (Fig. 6d). The guidewire was then translated and given an “S”-shaped curve to enter the first branch (Fig. 6e). Next, the guidewire was retracted and given an “S”-shaped curve with higher angulation at each joint to enter the second branch (Fig. 6f). Lastly, the guidewire was rotated to change the bending direction of the distal joint and translated to the base of the third branch (Fig. 6g) and translated further to the distal point of the branch (Fig. 6h).

To demonstrate the impact of stiffening during navigation, the guidewire was steered into the aortic arch and given a stiffening joint reference command, *θ*_*s*,*des*_, of ~32° as shown in Fig. 6i. The guidewire was then further inserted while the distal joint was commanded to bend ~85° under the following stiffness conditions: (1) with the natural stiffness of stiffening joint (Fig. 6j), (2) the desired stiffness of the stiffening joint set to *K*_*des*_ = 2*K*_*d*_ (Fig. 6k), and (3) the desired stiffness of the stiffening joint set to *K*_*des*_ = 5*K*_*d*_ (Fig. 6l). As shown in Fig. 6j, by actuating the distal joint of the guidewire, with the natural stiffness of the stiffening joint, we observe the motion of the stiffening joint as well. Thus, the stiffening joint significantly deviates from the setpoint of 32° (thereby demonstrating significant coupling between the distal joint and the proximal stiffening joint), causing contact on the interior walls of the phantom. The guidewire was then returned to the initial configuration. Next, using *K*_*des*_ = 2*K*_*d*_, the stiffening joint deviates ~19° from the setpoint of 32° (thereby demonstrating lesser inter-joint coupling between the distal joint and the proximal stiffening joint) during the process of bending the distal joint and advancing the guidewire. We further observe that the inter-joint segment moves closer to the centerline. The guidewire was then returned back to the initial configuration. Finally, using *K*_*des*_ = 5*K*_*d*_, the stiffening joint of the guidewire deviates ~2° from the setpoint of 32° (thereby demonstrating minimal inter-joint coupling between the distal joint and the proximal stiffening joint) when the guidewire was translated and the distal joint was bent.

## Discussion

The proposed robotically steerable guidewire with stiffness tuning capabilities using the motion control approach presented demonstrates the potential to replace the need for several guidewires of different stiffnesses in a given procedure. The additional capabilities can potentially facilitate the guidewire navigation in a relatively higher range of vasculatures as compared to a guidewire with only a steerable tip. The range of bending motions exhibited by the guidewire are still dependent on the machining parameters, which can be further tuned based on a given traversal path or for a specific endovascular procedure where stiffness variation could be desirable.

In the current study, the guidewire was fabricated using a 0.62 mm OD nitinol tube. The proposed guidewire exhibited a wide range of stiffness values of the stiffening joint, thereby demonstrating the high potential of the guidewire for use in clinical applications. Furthermore, unlike most studies on surgical robots which have only presented shape-locking mechanisms that translates to increasing the stiffness of the segment under consideration^26–29^, our work demonstrated both increasing and decreasing the stiffness of the segment under consideration. Additionally, the stiffness tuning capabilities were demonstrated for a sub-mm OD continuum robot within a sheath. Future studies will focus on further reducing the invasiveness of the proposed guidewire by utilizing nitinol tubes of smaller diameters. Existing studies have presented tendon-driven robotically steerable guidewires with OD as low as 0.4 mm^4^. However, miniaturization of the proposed guidewire will present challenges due to the sub-mm scale of the guidewire. The proposed guidewire can exhibit stiffness variation in only one fixed plane with respect to the bending plane of the steerable tip. While the bending plane of the steerable tip can be varied by rotating the guidewire about its central axis, the stiffness in the plane perpendicular to the bending plane remains a constant value with larger magnitude. Consequently, the robotic guidewire has limited bending capabilities in 3D-space and can only achieve variable stiffness within a single plane. Furthermore, the guidewire utilized in this work achieved a range of deflection that would not suffice in highly tortuous anatomy (e.g., neurointerventions). Future studies will address this issue by modifying the stiffness tuning mechanism to achieve stiffness variation in 3D-space utilizing a more compliant sheath and notch parameters designed for high angulation.

Furthermore, in current procedures for endovascular interventions, fluoroscopic imaging, which is the primary imaging modality for manual guidewire navigation, could be utilized for feedback control of robotically steerable guidewires. Since the imaging resolution of the X-ray machine is less than the CMOS camera used in this work, a more robust guidewire segmentation procedure would be required to achieve accurate segmentation of the markers (radiopaque for X-ray imaging). In fluoroscopic imaging, several challenges such as the similarity of the guidewire structure to the vessel walls, low contrast in imaging, and an overall small footprint of the guidewire may exist. Previous studies have developed different approaches for the aforementioned challenges ^22,53,54^. An accurate segmentation of the guidewire tip in 2D-space has been demonstrated by utilizing a cascaded convolutional neural network architecture^53^. In addition, the guidewire shape segmentation in 2D-space has been obtained using a semantic segmentation architecture based on MobileNetv2^22^. Zhao et al.^54^ propose a pyramid attention recurrent network (PAR-Net) for real-time guidewire segmentation in intraoperative fluoroscopic imaging. Similar network architectures can be integrated into the proposed control strategy for robust segmentation of the guidewire while using fluoroscopic feedback.

Moreover, while utilizing fluoroscopic feedback, continuous image feedback would not be possible as the X-ray machine is only used intermittently by the clinician to assist with guidewire navigation to avoid high radiation exposure to the personnel in the operating room. Therefore, the control algorithm used in this work would need to be modified to account for intermittent X-ray imaging. Furthermore, other shape reconstruction methods using intrinsic sensing modalities, such as fiber Bragg grating (FBG) sensors can be integrated along with fluoroscopic imaging to get a continuous feedback for closed-loop control of the system. This approach is hypothesized to provide sufficient feedback, with minimal noise, for developing a device capable of 3D stiffness control. Finally, the current clinical practices often utilize teleoperation wherein the operator is in the control loop to ensure safety of such model and feedback based control approaches. Our future work will also focus on developing intuitive control for teleoperation of the proposed guidewire for studies in an animal model.

## Methods

### Guidewire fabrication

The guidewire comprises two laser micromachined segments: a proximal segment and a distal segment. The distal segment of the guidewire comprising the steerable tip was designed to generate unidirectional planar motion, whereas the proximal end comprising the stiffening joint, was designed to generate bidirectional planar motion. Since the guidewire can be rotated about its central axis, unidirectional planar motion at the steerable tip was deemed to be sufficient as this design choice simplifies the overall mechanism of the steerable tip. Additionally, while bidirectional planar motion can be integrated at the tip, this would require more than one tendon to be attached to the guidewire tip. Routing more than one tendon through the inner channel of the tube may result in unwanted interaction between the tendons, consequently generating undesirable motion. The stiffening joint was designed to generate bidirectional motion to actively control the deflection of the stiffening joint without under-actuation, to achieve variable stiffening.

Several previous studies have demonstrated the use of laser micromachining for realizing compliant joints for continuum robots. The femtosecond laser used to micromachine the guidewire segments minimizes the heat-affected-zone (HAZ), and thus ensures that there is no accidental heat treatment of nitinol during the machining process^13^. The commonly employed micromachining patterns to generate planar bending motion include UAN, BSN, and the bidirectional asymmetric notch (BAN) patterns. While the UAN pattern facilitates bending in one direction, the BSN and BAN patterns enable bidirectional planar bending motion. The UAN pattern was utilized to realize a steerable tip in the proposed guidewire design because of its ability to provide an acceptable amount of stiffness and generate unidirectional motion. In contrast, a BSN pattern was employed to realize the stiffening joint due to its relatively high stiffness as compared to the other two notch patterns. The machining parameters (e.g., laser power, cutting speed) for the two selected patterns were tuned to achieve minimal precurvature in the segments after laser micromachining due to potential asymmetric heating, especially for the UAN pattern. These parameters were selected heuristically based on previous work from our laboratory^4,51^. The tendons to control the BSN pattern were routed along the outer surface of the tube, thereby causing minimal interaction between the distal and proximal segment tendons. To further ensure adequate alignment of the tendons with respect to the BSN pattern of the proximal segment, routing plates were utilized. The routing plates were laser micromachined on the femtosecond laser out of a 0.30 mm thick nitinol sheet. A routing plate was attached to every other unmachined segment between the notches of proximal segment (32 plates total) using J-B Weld KwikWeld Steel Reinforced Epoxy (J-B Weld, Sulphur Springs, TX, USA). The epoxy was applied to the unmachined segment using a 0.127 mm nitinol wire and allowed to cure overnight. In addition to preventing unwanted tendon interactions, the routing plates ensure the tendons closely follow the curvature of the proximal segment while undergoing deformation. A table summarizing all parameters of the proposed guidewire is shown in Table 2.

**Table 2:** Machining specifications of guidewire prototype

Component dimensions (mm)			
Total Length		749.3	
Total Steerable length		92.0	
NiTi Tube OD		0.62	
NiTi Tube ID		0.52	
Sheath OD		1.06	
Sheath ID		1.01	
Stiffening Tendon OD		0.076	
Distal Tendon OD		0.152	
Stiffening Joint Length, *l*_*s*_		35.72	
Stiffening Joint Notch Width, *h*_*s*_		0.315	
Stiffening Joint Notch Spacing, *c*_*s*_		0.285	
Stiffening Joint Notch Depth, *d*_*s*_		0.167	
Distal Joint Length, *l*_*d*_		35.72	
Distal Joint Notch Width, *h*_*d*_		0.315	
Distal Joint Notch Spacing, *c*_*d*_		0.285	
Distal Joint Notch Depth, *d*_*d*_		0.403	
Unmachined Segment Length, *l*_*m*_		20.0	

### Modeling

The mapping between the deflection of each joint from each tendon stroke is the combination of a kinematic term, *L*^*kin*^, and a tendon elongation term, *L*^*e*^, given by^13^:

4 $$X={L}^{kin}\left(q\right)+{L}^{e}\left(q\right)$$

where *X* refers to the required tendon stroke and *q* = ${\left[{\theta }_{d},{\theta }_{s}\right]}^{T}$. The required tendon stroke depends primarily on the material properties and notch parameters. The cross-sectional area of the distal joint notch, *A*_*d*_, is given by:

5 $${A}_{d}=\frac{\phi }{2}\left({r}_{o}^{2}-{r}_{i}^{2}\right)$$

The second moment of area of the distal joint notch, *I*_*d*_, is given by^55^:

6 $${I}_{d}=\frac{\left({r}_{o}^{4}-{r}_{i}^{4}\right)\left(\phi +\sin \phi \right)}{8}-\frac{8{\sin }^{2}\left(\frac{\phi }{2}\right){\left({r}_{o}^{3}-{r}_{i}^{3}\right)}^{2}}{9\phi \left({r}_{o}^{2}-{r}_{i}^{2}\right)}$$

Similarly, the cross-sectional area of the stiffening joint notch, *A*_*s*_, is given by:

7 $${A}_{s}=\left(\pi -\psi \right)\left({r}_{o}^{2}-{r}_{i}^{2}\right)$$

and the second moment of area of the stiffening joint notch, *I*_*s*_, is given by^11^:

8 $${I}_{s}=\frac{\pi }{4}\left({r}_{o}^{4}-{r}_{i}^{4}\right)-\frac{1}{4}\left({r}_{o}^{4}-{r}_{i}^{4}\right)\left(\psi +\sin \left(\psi \right)\right)$$

**Fig. 7:**
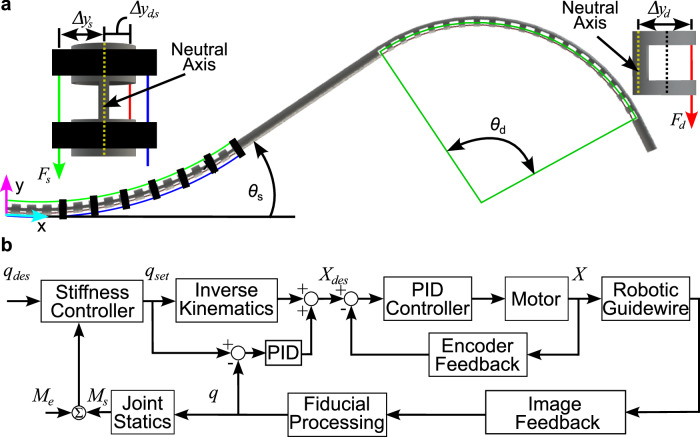
Overview of the model parameters and control strategy. **a** Schematic depicting the system parameters of the proposed robotically steerable guidewire. **b** Block diagram depicting the proposed control architecture for the guidewire.

Since the deflection of the joints are coupled together, the kinematic term for the distal joint, ${L}_{d}^{kin}\left({\theta }_{d},{\theta }_{s}\right)$, is given by:

9 $${L}_{d}^{kin}\left({\theta }_{d},{\theta }_{s}\right)=\Delta {y}_{d}{\theta }_{d}+\Delta {y}_{d,s}{\theta }_{s}$$

where Δ*y*_*d*,*s*_ is the distance from the center of the tube to the line of action of the distal tendon, and Δ*y*_*d*_ is the distance between the neutral axis of the distal joint and the distal tendon location (see Fig. 7a) given by:

10 $${\Delta} y_d = \underbrace{\frac{4\sin{\frac{\phi}{2}}\left(r_{o}^3-r_{i}^3\right)}{3\phi\left(r_{o}^2-r_{i}^2\right)}}_{{{\text{Neutral}}\,{\text{Axis}}\,{\text{Location}}}} {+} r_i -r_{{td}}$$

where *r*_*td*_ is the distal tendon radius. The moment-curvature relationship^23^ for the distal joint is given by:

11 $$\frac{{\theta }_{d}}{{l}_{d}}=\frac{{F}_{d}({\theta }_{d})\Delta {y}_{d}}{E{I}_{d}+{E}_{sh}{I}_{sh}}$$

where *F*_*d*_ is the force applied to the distal tendon, *E* is the Young’s modulus of the tube, *E*_*sh*_ is the Young’s modulus of the external sheath, and *I*_*sh*_ is the second moment of area of the sheath given by:

12 $${I}_{sh}=\frac{\pi }{4}\left({r}_{o,sh}^{4}-{r}_{i,sh}^{4}\right)$$

where *r*_*o*,*sh*_ and *r*_*i*,*sh*_ are the outer and inner radii of the sheath, respectively. The tendon elongation term, ${L}^{e}\left({\theta }_{d}\right)$, is determined by considering the applied tension to the tendon and is given by:

13 $${L}^{e}\left({\theta }_{d}\right)=\frac{{\eta }_{d}{F}_{d}\left({\theta }_{d}\right){L}_{d,0}}{{E}_{td}\pi {r}_{td}^{2}}$$

where *L*_*d*,0_ is the undeformed length of the distal tendon, *E*_*td*_ is the Young’s modulus of the distal tendon, and *η*_*d*_ is a friction loss term resulting from contact with the inner walls of the tube^4^. As a result, the tendon elongation term for a given joint deflection is given by:

14 $${L}^{e}\left({\theta }_{d}\right)={\eta }_{d}\frac{\left(E{I}_{d}+{E}_{sh}{I}_{sh}\right){L}_{d,0}}{\Delta {y}_{d}{E}_{td}\pi {r}_{td}^{2}{l}_{d}}{\theta }_{d}$$

where *l*_*d*_ is the length of the distal joint. The kinematic term for the stiffening joint is given by:

15 $${L}_{s}^{kin}\left({\theta }_{s}\right)=\Delta {y}_{s}{\theta }_{s}$$

where Δ*y*_*s*_ is the distance from the central axis of the stiffening joint to the location where the stiffening tendons are attached. Following the approach above, the elongation due to the stiffening joint will consider the moments applied from both the distal and stiffening tendons, where the moment-curvature relationship is given by:

16 $$\frac{{\theta }_{s}}{{l}_{s}}=\frac{{\eta }_{d,s}{F}_{d}\left({\theta }_{d}\right)\Delta {y}_{d,s}+{F}_{s}\left({\theta }_{s}\right)\Delta {y}_{s}}{E{I}_{s}+{E}_{sh}{I}_{sh}}$$

where *l*_*s*_ is the length of the stiffening joint, *F*_*s*_ is the stiffening tendon force, *η*_*d*,*s*_ accounts for friction losses between the stiffening joint and the distal joint, and *e*^*μα*^ is a capstan friction model to account for actuation system friction where *μ* is the friction coefficient between the nitinol tendon and the pulley, and *α* is the wrapping angle around the pulley. Similar to the distal joint, the moment-curvature relationship can be substituted into the tendon strain equation to determine the elongation term and is given by:

17 $${L}^{e}\left(q\right)=\frac{{\eta }_{s}{e}^{\mu \alpha }{L}_{s,0}}{\Delta {y}_{s}{E}_{ts}\pi {r}_{ts}^{2}}\left(\frac{\left(E{I}_{s}+{E}_{sh}{I}_{sh}\right){\theta }_{s}}{{l}_{s}}-\frac{{\eta }_{d,s}\left(E{I}_{d}+{E}_{sh}{I}_{sh}\right)\Delta {y}_{d,s}{\theta }_{d}}{\Delta {y}_{d}{l}_{d}}\right)$$

where *E*_*ts*_ is the Young’s modulus of the stiffening joint tendons, and *η*_s_ is a friction loss term due to wall contact. The coupled system is governed by a set of equations that are linear with respect to the joint deflections and can therefore be written as:

18 $$\left[\begin{array}{l} X_d \\ X_s\end{array}\right] = A\underbrace{\left[\begin{array}{ll}\theta_d \\ \theta_s \end{array}\right]}_{q}$$

where:

19 $$A=\left[\begin{array}{cc}\frac{{\eta }_{d}\left(E{I}_{d}+{E}_{sh}{I}_{sh}\right){L}_{d,0}}{\Delta {y}_{d}{E}_{t}\pi {r}_{td}^{2}{l}_{d}}+\Delta {y}_{d}&\Delta {y}_{d,s}\\ \frac{-{\eta }_{d,s}{\eta }_{s}{e}^{\mu\alpha }\left(E{I}_{d}+{E}_{sh}{I}_{sh}\right){L}_{s,0}\Delta {y}_{d,s}}{{E}_{ts}{l}_{d}\pi {r}_{ts}^{2}\Delta {y}_{d}\Delta {y}_{s}}&\frac{{\eta }_{s}{e}^{\mu\alpha }\left(E{I}_{s}+{E}_{sh}{I}_{sh}\right){L}_{s,0}}{{E}_{ts}{l}_{s}\pi {r}_{ts}^{2}\Delta {y}_{s}}+\Delta {y}_{s}\end{array}\right]$$

**Table 3:** Identified system parameters

Component	Value	Units
Young’s modulus of the tube, *E*	51.8	GPa
Young’s modulus of the sheath, *E*_*sh*_	3.6	GPa
Young’s modulus of the tendon of the stiffening joint, *E*_*ts*_	48.2	GPa
Young’s modulus of the tendon of the distal joint, *E*_*td*_	30	GPa
Inverse of the friction loss from the distal segment to the actuation system, *η*_*d*_	1.006	-
Inverse of the friction loss from the stiffening segment to the actuation system, *η*_*s*_	1.48	–
Inverse of the friction loss from the distal segment to the stiffening segment, *η*_*d*,*s*_	1.002	–
Total wrapping angle of tendon pulleys, *α*	15	°
Coefficient of friction between nitinol tendons and pulleys, *μ*	0.199	–

The parameters identified based on experiments.

As a result, a desired configuration of joint angles can be used to determine the required tendon stroke. Furthermore, *A* is non-singular and hence we can derive the deflection angle from the tendon stroke by the following relation:

20 $$\left[\begin{array}{c}{\theta }_{d}\\ {\theta }_{s}\end{array}\right]={A}^{-1}\left[\begin{array}{c}{X}_{d}\\ {X}_{s}\end{array}\right]$$

To determine the Young’s modulus of the sheath and nitinol tube, each tube was cantilevered and imaged. The tip displacement was compared to a simple cantilever model using a density of 6450 kg/m^3^ ^56^ and 1160 kg/m^3^ for the nitinol and polyimide tubes, respectively. The resulting Young’s modulus for the nitinol tube, *E*, and the polyimide tube, *E*_*sh*_, were determined to be 51.8 GPa and 3.6 GPa, respectively. The tendon of the stiffening joint (*r*_*ts*_ = 0.038 mm) was loaded until failure using a Mark-10 (Mark-10 F505, Mark-10 Corporation, NY, USA) resulting in *E*_*ts*_ = 48.2 GPa. The Young’s modulus of the distal tendon (*r*_*ts*_ = 0.076 mm) was experimentally determined to be *E*_*td*_ = 30 GPa. The model parameters identified through experiments are presented in Table 3.

### Stiffness controller

The distal and stiffening joints can be steered autonomously or by an operator to desired bending angles of *θ*_*d*,*des*_ and *θ*_*s*,*des*_, compactly written as *q*_*des*_. In this work, the goal of the stiffness control is to generate reference joint angle values that deviate from the desired configuration in response to distal joint actuation. The incorporation of external stimuli could be included into the model, and thus the control law, using a priori knowledge (e.g., location) of external loads^57^ or through force estimation and localization approaches^11,58^. The deviation from the configuration is selected to emulate the behavior of a joint with desired stiffness, *K*_*des*_. As a result, the setpoint for the stiffening joint is given by:

21 $${\theta }_{s,set}={\theta }_{s,des}+\Delta {\theta }_{s}(q)$$

where $\Delta {\theta }_{s}(q)$ is determined by a user-defined feedback law and *θ*_*s*,*set*_ is the setpoint passed to the joint controllers. In this work, we determine the Δ*θ*_*s*_ to achieve varying mechanical coupling between the joints by choosing a desired stiffness of the stiffening segment. For example, if the desired stiffness is selected to be increasingly less than the distal joint stiffness, the stiffening joint would deflect increasingly more when the distal joint is actuated due to the forces the distal tendon imparts on the stiffening segment. The joint coupling is given by the ratio of the deflection of each joint, determined by the moment-curvature relationship, assuming only a distal force is being applied as a result of the distal tendon displacement, *X*_*d*_. By combining Eq. (11) and Eq. (16), the joint coupling is given by:

22 $$\frac{{\theta }_{s}}{{\theta }_{d}}=\frac{{l}_{s}\Delta {y}_{d,s}{\eta }_{d,s}\left(E{I}_{d}+{E}_{sh}{I}_{sh}\right)}{{l}_{d}\Delta {y}_{s}\left(E{I}_{s}+{E}_{sh}{I}_{sh}\right)}$$

The natural stiffness of the stiffening joint (*EI*_*s*_ + *E*_*sh*_*I*_*sh*_) in Eq. (22) can be replaced by the desired stiffness, *K*_*des*_. The resulting joint coupling is given by:

23 $$\frac{{\theta }_{s}}{{\theta }_{d}}=\frac{{l}_{s}\Delta {y}_{d,s}{\eta }_{d,s}\left(E{I}_{d}+{E}_{sh}{I}_{sh}\right)}{{l}_{d}\Delta {y}_{s}{K}_{des}}$$

As a result, the setpoint for the joint angles including a desired stiffening joint stiffness responding to the actuation of the distal joint is given by:

24 $$\theta_{s,set} = \theta_{s,des} {+} \underbrace{K_{des}^{-1}\left(\frac{l_s{\Delta} y_{d,s}\eta_{d,s}\left(EI_d{+}E_{sh}I_{sh}\right)}{l_d{\Delta} y_s}\theta_d\right)}_{{\Delta} \theta_s}$$

Considering a joint with uniform notch parameters, the control law presented will result in variations in the stiffening joint motion that are linearly proportional to the distal joint motion. The desired distal joint angle and the generated stiffening setpoint are then passed to the inverse model (Eq. (18)) to generate the desired tendon stroke. The error measured from imaging is used to compensate for phenomenon, such as hysteresis, which is not accounted for in the model. The error is passed through a PID controller and added to the references provided by the inverse model. The tendon stroke commands are then converted into motor rotations through the lead screw pitch and controlled using PID control with encoder feedback to precisely translate each tendon. A block diagram indicating the flow of information to control the stiffness of the stiffening joint of the robotically steerable guidewire is shown in Fig. 7b.

## Data Availability

The datasets used and/or analyzed during the current study are available from the corresponding author upon reasonable request.
